# Shedding Light on Depth: A Meta-analysis of Light Versus Deep Anaesthesia and Postoperative Neurocognitive Outcomes in Elderly Surgical Patients

**DOI:** 10.7759/cureus.98647

**Published:** 2025-12-07

**Authors:** Hosam Hadi Hassan Awaji, Norah Alsulaiman, Mshary S Alshahrani, Falwah S Alsalman, Abdulaziz I Alassaf, Rakan H Alabdulali, Abdulrahman J Alfaifi, Abdullah I Ghabban, Sayer H Aljuaid

**Affiliations:** 1 Preventive Medicine, North West Armed Forces Hospital, Tabuk, SAU; 2 Rehabilitation, National Guard Hospital, Riyadh, SAU; 3 Anesthesiology, King Khalid University College of Medicine, Abha, SAU; 4 Medicine, Alfaisal University College of Medicine, Riyadh, SAU; 5 Medicine, King Saud bin Abdulaziz University for Health Sciences College of Medicine, Riyadh, SAU; 6 General Practice, Alfaisal University College of Medicine, Riyadh, SAU; 7 Family Medicine, Taif University, Taif, SAU; 8 Emergency Medicine, King Fahad Specialist Hospital, Tabuk, SAU

**Keywords:** deep anaesthesia, light anaesthesia, pacu discharge, postoperative cognitive dysfunction, postoperative delirium

## Abstract

Postoperative delirium (POD) and postoperative cognitive dysfunction (POCD) are common and serious complications in elderly surgical patients, especially after prolonged procedures. Recent evidence suggests that the depth of anaesthesia may influence the risk of these adverse outcomes. This meta-analysis examined whether light versus deep anaesthesia affects the incidence of POD and POCD, as well as recovery measures such as time to post-anaesthesia care unit (PACU) discharge and length of hospital stay in elderly patients. A systematic search identified randomized controlled trials (RCTs) published in English up to May 24, 2025, involving patients aged ≥60 years undergoing major surgery. The outcomes evaluated were POD, POCD, PACU discharge time, and hospital stay. Meta-analyses were conducted using random- or fixed-effects models based on heterogeneity. Eight RCTs with a total of 1,957 elderly patients were included. Light anaesthesia was associated with a significantly lower incidence of POD (OR = 0.52; 95% CI: 0.41-0.66; P < 0.00001; I² = 0%). The incidence of POCD was also reduced with light anaesthesia (OR = 0.55; 95% CI: 0.39-0.77; P = 0.0005; I² = 15%). Time to PACU discharge was shorter under light anaesthesia (mean difference (MD) = -9.43 minutes; 95% CI: -12.87 to -5.99; P < 0.00001; I² = 66%). Although hospital stay showed a trend favouring light anaesthesia (MD = -0.81 days; 95% CI: -1.85 to 0.24; P = 0.13), this result was not statistically significant (I² = 89%). Overall, light anaesthesia appears to reduce the incidence of POD and POCD and is associated with faster early recovery in elderly surgical patients. The observed trend toward shorter hospital stays did not reach significance. These findings indicate potential benefits of lighter anaesthetic strategies, though they should not be interpreted as evidence of direct causation, as other factors such as anaesthetic drug type and surgical invasiveness may also contribute.

## Introduction and background

Postoperative delirium (POD) and postoperative cognitive dysfunction (POCD) are common and serious complications in older adults undergoing major surgery. These neurocognitive disorders are linked to higher morbidity, longer hospital stays, functional decline, and long-term cognitive impairment. As global populations age and surgical procedures become more common in elderly individuals, optimizing perioperative strategies to prevent these complications has become increasingly important [1].

One intraoperative factor of interest is the depth of anaesthesia. Anaesthetic depth can vary between lighter and deeper levels and is typically guided by processed EEG-based monitoring systems such as the Bispectral Index (BIS) [2]. Other commercially available monitors include the Patient State Index (PSI), Entropy, and Narcotrend, each using different algorithms and calibration methods. To maintain consistency and comparability across trials, this review focuses only on BIS-based studies [3]. While deeper anaesthesia may help achieve patient immobility and stable operating conditions, concerns have been raised that excessive depth could worsen postoperative neurocognitive outcomes. Conversely, lighter anaesthetic levels may reduce potential neurotoxicity but raise the risk of intraoperative awareness or hemodynamic instability [4].

Light and deep anaesthesia can generally be distinguished by their effects on brain activity and patient responsiveness, often quantified using BIS values: light anaesthesia typically corresponds to BIS levels of 50-60, while deep anaesthesia targets values below 40 [5]. Although deep anaesthesia provides immobility and minimizes awareness, it has been associated with greater cerebral suppression, hypotension, and reduced cerebral perfusion, factors speculated to contribute to POD and POCD [6]. In contrast, lighter anaesthesia may better preserve cerebral physiology but may also risk inadequate sedation or patient movement. Achieving an optimal balance is particularly important in older adults, who are more vulnerable to neurological injury [7].

Although several randomized trials suggest an association between deeper anaesthesia and higher rates of POD or POCD, the evidence remains inconsistent and observational in nature, with many studies reporting no significant differences between light and deep targets. Some findings support reduced neurocognitive complications with lighter anaesthesia, while others show neutral or inconclusive results. Anaesthetic depth may also influence other postoperative recovery measures, such as post-anaesthesia care unit (PACU) discharge time and length of hospital stay [8,9].

Among the available EEG-derived depth monitors, the BIS is the most extensively validated in elderly surgical patients and has been used in most RCTs evaluating anaesthetic depth. Because of differences in algorithms, calibration, and sensitivity across devices, combining trials using different monitors could introduce substantial heterogeneity. Therefore, this meta-analysis includes only BIS-based studies to ensure methodological consistency [10].

Given the ongoing uncertainty and the clinical importance of postoperative neurocognitive disorders in older patients, a consolidated evaluation of existing evidence is needed. The aim of this meta-analysis is to assess the impact of light versus deep anaesthesia on the incidence of POD and POCD, as well as secondary outcomes such as PACU discharge time and hospital stay, in elderly individuals undergoing major surgery. By synthesizing data from randomized controlled trials (RCTs), this study seeks to support evidence-based anaesthetic management and improve perioperative care in this vulnerable population.

## Review

Methods

The conduct and reporting of this meta-analysis adhered to the Cochrane Handbook for Systematic Reviews of Interventions (Version 6) and followed the Preferred Reporting Items for Systematic Reviews and Meta-Analyses (PRISMA) guidelines [11].

The Research Question

What are the effects of light anaesthesia compared with deep anaesthesia on POD, POCD, PACU discharge time, and length of hospital stay in older adults undergoing major surgery?

Research Aims and Objectives

This study aims to systematically synthesize and compare evidence from RCTs evaluating the impact of light versus deep anaesthesia on POD, POCD, and recovery outcomes in elderly patients undergoing major surgery; to determine the incidence of POD in elderly patients receiving light compared with deep anaesthesia during major surgery; to compare time to discharge from the PACU between the two anaesthetic depth groups; to compare hospital stay in older adults receiving light anaesthesia with those receiving deep anaesthesia; and to explore potential sources of heterogeneity, including the type of surgery, anaesthetic drugs used, and methods of depth monitoring in the included RCTs.

Inclusion Criteria

This meta-analysis includes RCTs published from 2000 to May 24, 2025, that compared light versus deep anaesthesia in older patients undergoing major surgery. Only full-text, peer-reviewed articles published in English were included. Studies were eligible if they used validated monitoring tools to determine anaesthesia depth (e.g., BIS or other EEG-based monitors such as PSI, Entropy, Narcotrend, or raw EEG-based monitoring).

Participants

Eligible trials included older patients, generally aged >60 years, undergoing major surgery under general anaesthesia. Surgeries included, but were not limited to, cardiac, orthopaedic, abdominal, and oncological procedures. Patients were required to be cognitively intact at baseline and free from severe, diagnosed dementia.

Interventions

Intervention group (light anaesthesia): The intervention group consisted of patients receiving general anaesthesia titrated to lighter depths, typically targeting BIS values of 50-60 or equivalent.

Control group (deep anaesthesia): The control group included patients receiving deeper levels of general anaesthesia, generally targeting BIS values <40 or equivalent.

Both groups received standard perioperative care, including analgesia and intraoperative monitoring, according to institutional protocols.

Although the intended targets were BIS 50-60 for light anaesthesia and <40 for deep anaesthesia, some included RCTs used slightly different BIS ranges (e.g., 35, 40-55, or 40-60). A few trials titrated anaesthetic depth using alternative EEG-based metrics or cerebral oximetry-guided optimization. To preserve consistency with each trial’s randomization and avoid excluding eligible studies, groups were classified according to the trial-defined lighter and deeper anaesthesia targets.

Exclusion Criteria

Studies were excluded if they were observational studies, case series, or reviews; enrolled non-elderly participants (<60 years) or mixed-age populations without separate analysis for older adults; did not report outcomes for POD, POCD, PACU discharge time, or hospital length of stay; lacked explicit definitions or monitoring of anaesthesia depth; included patients with pre-existing severe cognitive impairment or diagnosed dementia; used regional anaesthesia or sedation alone without general anaesthesia; and were published in languages other than English without an approved translation.

Search Strategy

Electronic searches: MEDLINE/PubMed, Cochrane Central Register of Controlled Trials (CENTRAL), Web of Science, ProQuest, and Scopus were searched for eligible studies. The search was set for all articles published in English from inception until May 24, 2025. using the following search terms: ("aged" OR "elderly" OR "older adults" OR "geriatric") AND ("depth of anaesthesia" OR "depth of anesthesia" OR "Bispectral index" OR BIS OR "light anesthesia" OR "deep anesthesia") AND ("postoperative delirium" OR "delirium" OR "emergence delirium" OR "acute confusion") AND ("major surgery" OR "orthopedic surgery" OR "abdominal surgery" OR "non-cardiac surgery"). Appendix A summarizes the used search terms for each database and the count of search results.

Other resources: The first reviewer searched within the reference lists of obtained articles for other potentially relevant studies that were not retrieved by electronic search.

Selection of Studies

The first reviewer screened the retrieved reports for eligibility through title and abstract and full-text screening. The second reviewer checked the retrieved studies, and discrepancies were solved through discussion with a third reviewer.

Data Extraction

The first reviewer carried out data extraction from the included studies using a standardized data sheet that included (a) the study’s characteristics (author, year, country, study design); (b) patients’ characteristics (sample size, studied groups, age at the time of intervention, sex, ASA physical status); (c) intervention and control details (type of surgery, anaesthesia type, anaesthetic agents, use of nitrous oxide, concomitant medications, opioid use, surgery duration, anaesthesia duration, delirium assessment tool, POD subtypes reported, depth of anaesthesia assessment tool, follow-up duration) , and (e) the outcomes (POD incidence, POCD incidence, time to PACU discharge (minutes), length of hospital stay (days)). The second reviewer checked the collected data for consistency and clarity, with any disagreements resolved by a third reviewer.

Measured Outcomes

The measured outcomes included the incidence of POD, defined as acute disturbances in attention and cognition within days after surgery and assessed using standardized tools such as the Confusion Assessment Method (CAM); the incidence of POCD, defined as a measurable decline in cognitive function after surgery assessed via neuropsychological testing; time to PACU discharge, measured in minutes from the end of surgery based on standard discharge criteria; and length of hospital stay, measured in days from the day of surgery until discharge.

Assessment of the Risk of Bias in Included Studies

The risk of bias (ROB) in the included studies was assessed using the National Institute for Health and Care Excellence (NICE) checklists for randomized controlled clinical trials [12].

Data Synthesis

**Figure 1:**
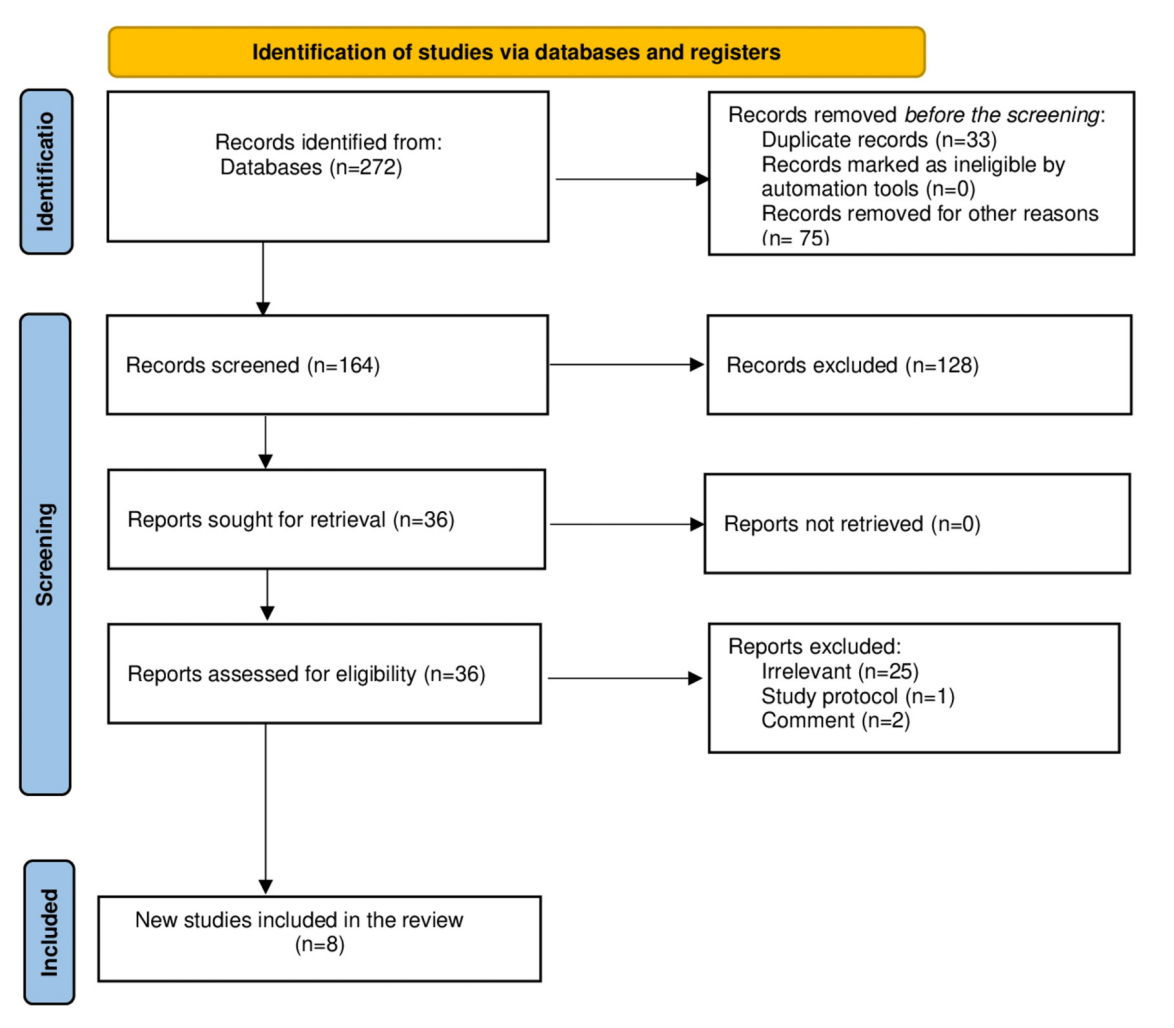
PRISMA flowchart of the included studies

Initially, 272 records were retrieved from electronic database searches. After removing duplicates and excluded studies, 36 studies were finally eligible, of which 8 studies (1,957 patients) were included [13-20] (Table 1). The 28 excluded studies were either irrelevant (n = 25), study protocol (n = 1), comment (n = 2). The PRISMA [21] flowchart of the study selection process is provided in Figure 1.

**Table 1:** Summary of the included studies NM: not mentioned.

Author	Year	Country	Study design	Sample size	Studied groups (intervention group vs. control group)	Mean age/age range	% female	ASA physical status	Type of surgery	Elective or emergency surgery	Anaesthesia type	Anaesthetic agents	Use of nitrous oxide	Concomitant medications	Opioid use	Surgery duration (minutes)	Anaesthesia duration (minutes)	Delirium assessment tool	POD subtypes reported	Depth of anaesthesia assessment tool	Follow-up period	Outcomes	Key results
Chan et al. [13]	2013	Hong Kong	RCT	921	BIS-guided (40-60) vs. routine care	68.1 ± 8.2 (BIS), 67.6 ± 8.3 (control)	37.8% (BIS), 39.6% (control)	ASA 1-2: 82.8% (BIS), 84.5% (control)	Major noncardiac	Elective	General	Propofol + volatile agents	53.5% (BIS), 57.4% (control)	NM	Fentanyl + morphine (similar doses between groups)	2.1 ± 1.0 hours (BIS), 2.0 ± 1.1 hours (control)	NM	Confusion Assessment Method (CAM)	NM	BIS monitor	3 months	Delirium incidence, time to eye opening, PACU discharge, hospital stay	Delirium incidence: 15.6% (BIS) vs. 24.1% (control) (P = 0.01); time to eye opening: 10 vs. 15 min (P < 0.001); PACU discharge: 80 vs. 92 min (P < 0.001); hospital stay: 7 vs. 8 days (P=0.98)
Evered et al. [14]	2021	Australia, China (including Hong Kong SAR), USA	Multicentre randomized clinical trial (sub-study of the BALANCED trial)	515 patients (253 BIS 50, 262 BIS 35)	BIS 50 (light anaesthesia) vs. BIS 35 (deep anaesthesia)	70.8-71.1 years; participants aged ≥60 years	35#-36%	98% ASA 3, 2% ASA 4	Major surgery (intra-abdominal, orthopaedic, spinal, thoracic, vascular)	98.8%$-99% elective, 1.0%-1.2% emergency	Volatile-agent-based general anaesthesia	Volatile agents (e.g., sevoflurane/desflurane), propofol, midazolam, fentanyl, remifentanil, morphine, ketamine	Excluded	Inotropes/vasopressors (53% BIS 50 vs. 72% BIS 35), NSAIDs, tramadol, paracetamol	Fentanyl (76%-78%), remifentanil (35%-37%), morphine (37%-40%)	Median: 216-221 (IQR: 157-298)	Defined as time from BIS < 60 to agent discontinuation (exact duration not reported)	3D-CAM (98.5% assessments), CAM-ICU (1.5%)	Syndromal delirium (reduced in BIS 50) and sub-syndromal delirium (no difference)	BIS (bispectral index)	Delirium: 5 days post-op; cognitive assessments at discharge: 30 days, 1 year	Primary: POD incidence; secondary: mortality, cognitive decline, ICU admission, length of stay	POD incidence: 19% (BIS 50) vs. 28% (BIS 35) (OR 0.58, P = 0.010); better 1-year cognition in BIS 50 (AMTS ≤ 6: 9% vs. 20%, P < 0.001)
He Z et al. [15]	2023	China	Randomized controlled trial (RCT)	141 randomized, 125 analyzed (61 rEEG-guided, 64 routine care)	Intervention: rEEG-guided anaesthesia (targeting C-D sedation depth); control: routine care (clinical experience-guided)	Median 68 (rEEG) vs. 69 (control)	36% (rEEG) vs. 33% (control)	Mostly ASA II (56%-69%) and III (29%-44%)	Major abdominal (gastrointestinal, hepatobiliary-pancreatic)	Elective	Total intravenous anaesthesia (TIVA)	Propofol, remifentanil, sufentanil, midazolam, etomidate	not used	Cisatracurium, vasoactive drugs	Remifentanil (infusion), sufentanil (bolus)	Median 155-160 min	Median ~155 to 160 min	CAM tool	NM	Raw EEG (rEEG) with sedation stages (C-D)	30 days	Primary: postoperative complications (respiratory, cardiovascular, neurological, gastrointestinal); secondary: intraoperative burst suppression (BS), frontal alpha power	No difference in POD incidence (3% vs. 11%; *P > 0.05), reduced BS duration in rEEG group (*P < 0.05)
Kunst et al. [16]	2020	UK	RCT	82	BIS + cerebral oximetry optimization vs. control	71.6 ± 5.0 (intervention), 72.0 ± 4.3 (control)	21% (intervention), 15% (control)	NM	Elective coronary artery bypass grafting (CABG)	Elective	General	Isoflurane	Not used	NM	Remifentanil + morphine	86 vs. 76 min (CPB time, P = 0.02)	NM	CAM tool	NM	BIS + cerebral oximetry	6 weeks	Delirium incidence, hospital stay	Delirium incidence: 2.4% (intervention) vs. 20% (control) (P = 0.01); hospital stay: 8.2 vs. 7.4 days (P = 0.34)
Long et al. [17]	2024	China	Pilot randomized, double-blind, 2 × 2 factorial trial	80 patients randomized; 78 completed the trial	Dexmedetomidine + deep anaesthesia (n = 19), dexmedetomidine + light anaesthesia (n = 20), placebo + deep anaesthesia (n = 19), placebo + light anaesthesia (n = 20)	69.6 years (SD 4.6), age range: ≥60 years	38% (30 out of 78 patients)	Not specified in the abstract	Major noncardiac surgery	Not specified in the abstract	General anaesthesia	Dexmedetomidine infusion at 0.5 μg/kg/h or normal saline placebo	Not specified in the abstract	Not specified in the abstract	Not specified in the abstract	Not specified in the abstract	Not specified in the abstract	Not specified in the abstract	Not specified in the abstract	Light anaesthesia: BIS target of 55; deep anaesthesia: BIS target of 40	Twice-daily POD screening; duration not specified in the abstract	Primary outcome: feasibility of the study design; secondary outcomes: incidence of postoperative delirium (POD)	Overall POD incidence: 10 out of 78 patients (13%; 95% CI, 7%-22%); placebo group: 7 out of 39 patients (17.9%; 95% CI, 9%-32.7%); deep anaesthesia group: 7 out of 38 patients (18.4%; 95% CI, 9.2%-33.4%); estimated between-group difference for dexmedetomidine vs. placebo: -10% (95% CI, -28% to 7%); estimated between-group difference for light vs. deep anaesthesia: -11% (95% CI, -28% to 6%)
Sieber et al. [18]	2010	USA	Double-blind RCT	114 (57 vs. 57)	Light sedation (BIS ≥ 80) vs. deep sedation (BIS ~ 50)	81 years	70% (light), 75% (deep)	NM	Hip fracture repair	Emergency	Spinal anaesthesia with propofol sedation	Propofol, midazolam, fentanyl	Not used	Hydromorphone, morphine postoperatively	Postoperative hydromorphone/morphine	Light: 79 ±33; deep: 93 ±44	NM	Confusion Assessment Method (CAM)	NM	BIS	Hospitalization period	Delirium prevalence, days of delirium	Light sedation reduced delirium (19% vs. 40%, *P = 0.02)
Wong et al. [19]	2002	Canada	RCT	60 (31 vs. 29 after exclusions)	BIS-titrated vs. standard practice	70-71 years	32%-34%	ASA I-III	Elective orthopedic (knee/hip)	Elective	General anaesthesia	Isoflurane, nitrous oxide, propofol, fentanyl	Yes (60%-70%)	Rocuronium, midazolam	Intraoperative fentanyl, postoperative PCA morphine	90 ±16	120 ±17	Mini-Mental State Examination (MMSE), Trieger Dot Test,Digit Symbol Substitution Test (DSST)	NM	BIS	Up to 72 hours post-op	Recovery times, cognitive tests	Faster recovery in BIS group; no cognitive difference
Zhou et al. [20]	2018	China	RCT (double-blind)	81 analyzed (41 BIS-guided, 40 control)	Intervention: BIS-guided anaesthesia (target BIS 40-60); control: standard hemodynamic monitoring	68.3 ± 2.8 (BIS) vs. 68.9 ± 3.0 (control)	29.3% (BIS) vs. 32.5% (control)	ASA 1-3	Radical colon carcinoma resection	Elective	TIVA	Propofol, remifentanil, vecuronium	Not used	Sufentanil (postoperative analgesia)	Remifentanil (intraoperative), sufentanil (postoperative)	155-185 min	155-185 min	CAM tool	NM	BIS monitor	5 days postoperatively	Primary: attention network functions (alerting, orienting, executive control); secondary: POD incidence	Lower POD in BIS group (17.5% vs. 27.5%, *P < 0.001); reduced propofol/remifentanil doses in BIS group (*P < 0.001)

Statistical Analysis

Meta-analysis was performed using the Review Manager (RevMan), Version 5.4 [22]. For dichotomous outcomes, such as the incidence of POD and POCD, pooled ORs with corresponding 95% CIs were calculated. For continuous outcomes, including time to discharge from the PACU and length of hospital stay, pooled mean differences (MDs) with 95% CIs were computed. A random-effects model was employed to account for anticipated clinical and methodological heterogeneity among the included studies. Heterogeneity was evaluated using the I² statistic, with values greater than 50% considered indicative of substantial heterogeneity. Sensitivity analyses were performed by sequentially excluding individual studies to assess the robustness and stability of the pooled estimates. In cases of notable heterogeneity, specific studies contributing disproportionately to variability were identified and their exclusion tested for impact on overall results.

Results

This meta-analysis included eight RCTs [13-20], encompassing a total of 1,957 elderly patients, to evaluate the impact of anaesthetic depth (light versus deep anaesthesia) on the incidence of POD in older adults.

POD Incidence

**Figure 2:**
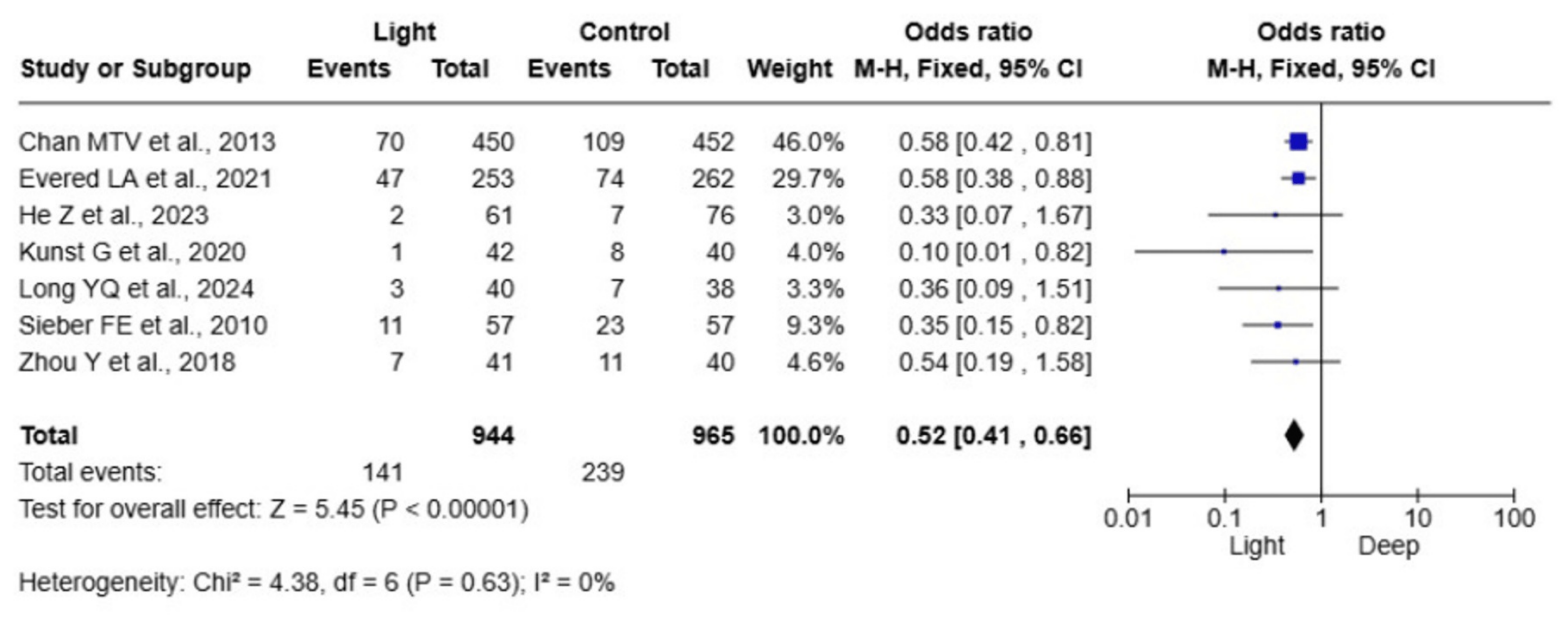
Forest plot comparing the incidence of postoperative delirium (POD) between light and deep anaesthesia

Seven studies [13-18,20] assessed the incidence of POD following light versus deep anaesthesia, encompassing 1,897 elderly patients. The pooled data demonstrated that light anaesthesia was associated with a significantly lower incidence of POD compared to deep anaesthesia (OR= 0.52; 95% CI: 0.41, 0.66; P < 0.00001). Heterogeneity among the included studies was low (I² = 0%), indicating consistent results across studies (Figure 2).

POCD Incidence

**Figure 3:**
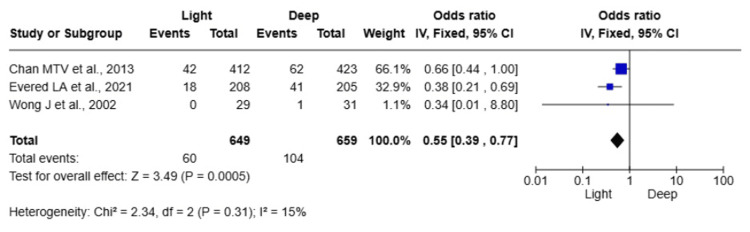
Forest plot comparing the incidence of postoperative cognitive dysfunction (POCD) between light and deep anaesthesia

Three studies [13,14,19] evaluated 1,308 elderly patients for the incidence of POCD. The pooled analysis revealed a significantly lower incidence of POCD in patients who received light anaesthesia compared to those who received deep anaesthesia (OR 0.55; 95% CI: 0.39, 0.77, P = 0.0005). Heterogeneity among the included studies was low (I² = 15%), suggesting consistent findings across studies (Figure 3). 

Time to PACU Discharge (Minutes)

**Figure 4:**
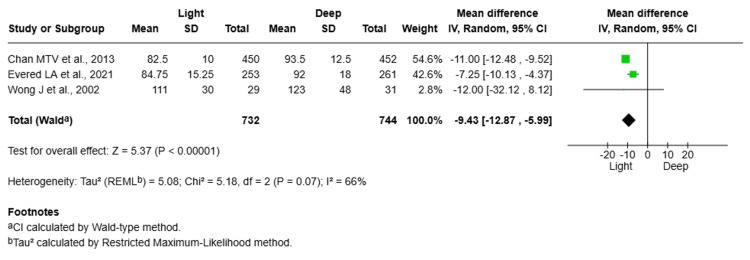
Forest plot comparing time to PACU discharge between light and deep anaesthesia

Three studies [13,14,19], including a total of 1,476 elderly patients, reported on time to discharge from the PACU. Patients in the light anaesthesia group had a significantly shorter time to PACU discharge compared to those in the deep anaesthesia group (pooled MD: -9.43 minutes, 95% CI: -12.87, -5.99; P < 0.00001). There was moderate heterogeneity across studies (I² = 66%). Sensitivity analysis showed that heterogeneity was mainly driven by the study by Wong et al. [19], which had a small sample size and a wide confidence interval; excluding this study effectively resolved the heterogeneity (Figure 4). 

Length of Hospital Stay (Days)

**Figure 5:**
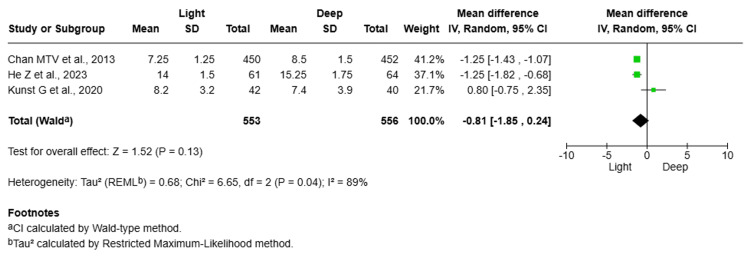
Forest plot comparing length of hospital stay between light and deep anaesthesia in patients with delirium

Three studies [13,15,16], comprising 1,109 elderly patients, assessed the length of hospital stay (days) following surgery under light and deep anaesthesia in elderly patients with delirium. The pooled MD was -0.81 days, favouring the light anaesthesia group, although this result did not reach statistical significance (MD = -0.81; 95% CI: -1.85, 0.24, P = 0.13). The heterogeneity was primarily driven by the study by Kunst et al. [16], which reported an MD favouring deep anaesthesia. Excluding Kunst et al. [16] from the analysis markedly reduced heterogeneity and supported a more consistent trend favouring light anaesthesia (Figure 5). 

Postoperative Analgesia

In the study by Wong et al. [19], patients received patient-controlled analgesia (PCA) with morphine, postoperatively; however, the authors reported no significant difference in postoperative cognitive test performance between groups and specifically noted that the administration of PCA morphine may have minimized any potential intergroup differences, though it was not analyzed as an independent risk factor. Equally, in the CODA Trial by Chan et al. [13], postoperative PCA morphine was given, but their multivariable analysis did not find intraoperative opioid dosage to be an independent predictor for either POCD at three months or POD. The BALANCED sub-study by Evered et al. [14] also did not find a significant association between intraoperative opioid administration and the incidence of POD in their analysis. Overall, while postoperative opioid-based analgesia was a consistent feature of the care provided, none of these studies reported a statistically significant increase in POD or POCD due to the analgesic regimen within the cohorts they analyzed.

Risk of Bias

The overall risk of bias among the included RCTs was generally low, with most studies demonstrating strong methodological rigor. Unfortunately, depth of anaesthesia was assessed using different methods across trials, and BIS-based studies used inconsistent cutoff values to define “light” versus “deep” anaesthesia. This introduces potential misclassification of the intervention and may bias the estimated effects.

**Figure 6:**
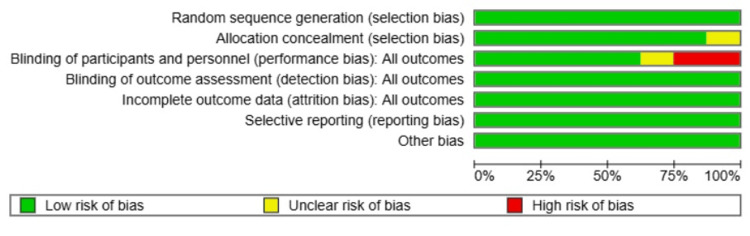
Risk of bias graph across all included studies

**Figure 7:**
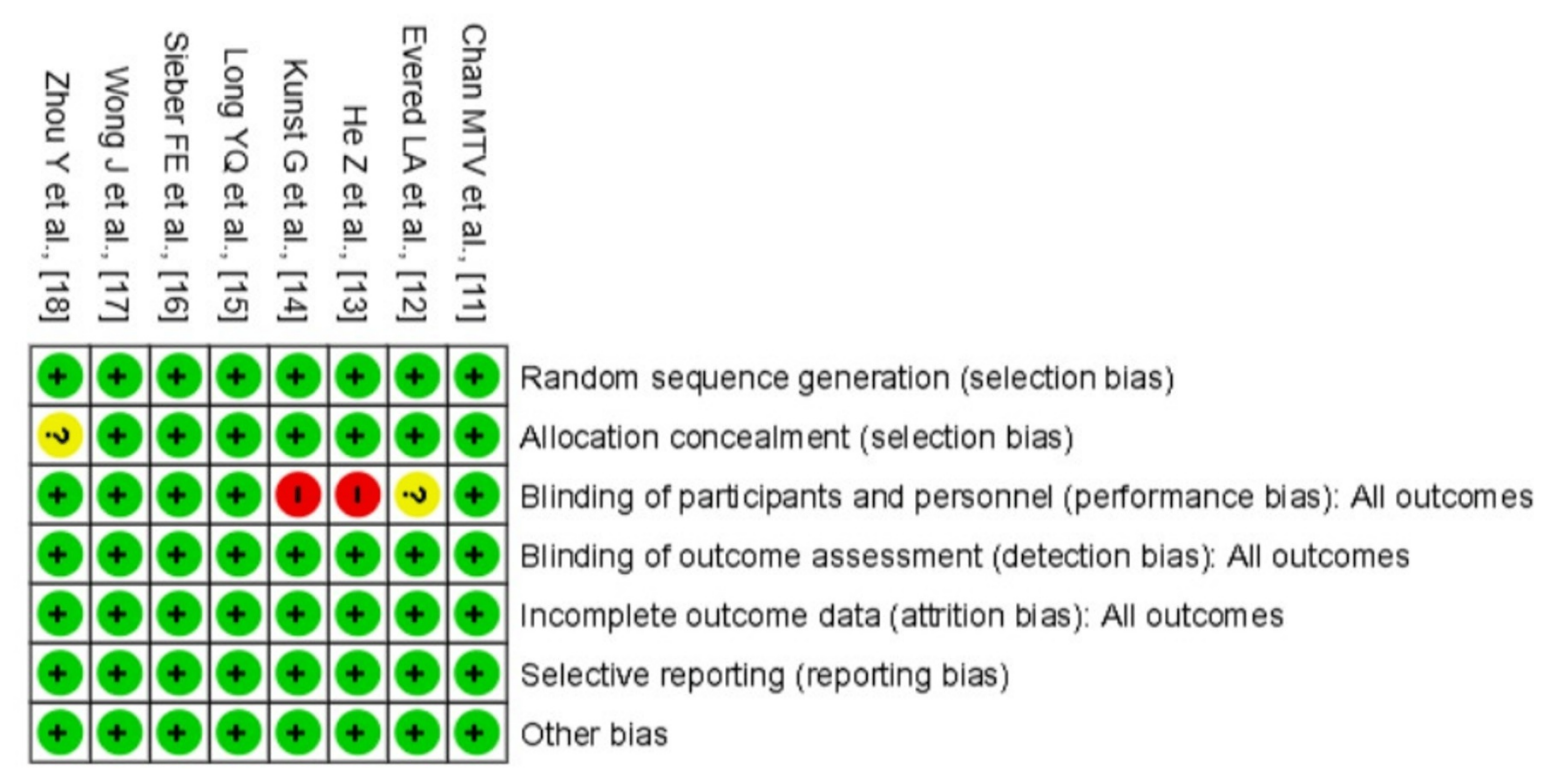
Risk of bias summary judgements for individual studies across seven domains

All studies adequately reported random sequence generation, and allocation concealment was clearly described in all but one study [20], which was rated as unclear due to insufficient reporting. Blinding of participants and personnel was maintained in the majority of studies; however, He et al. [15] and Kunst et al. [16] were judged to have a high risk of performance bias due to lack of blinding, while Evered et al. [14] were rated as unclear in this domain. Blinding of outcome assessment was consistently adequate across all studies, indicating low risk of detection bias. Most trials handled attrition well, with Evered et al. [14] excluding 32 patients due to missing baseline data (accounted for in the CONSORT flowchart) and judged to be low risk. No evidence of selective reporting was found in any of the studies. Minor concerns were noted under other biases, including the single-centre design of He et al. [15], small sample sizes in Wong et al. [19] and Zhou et al. [20], and possible learning effects in cognitive assessments in Wong et al. [19]. However, these were not deemed to significantly compromise study validity. Overall, despite isolated instances of high or unclear risk, particularly related to performance bias, the included studies were largely of high quality, supporting a moderate-to-high confidence in the meta-analysis findings (Figures 6, 7).

Discussion

POD and POCD are frequent disorders in elderly patients following major surgery. They are associated with increased morbidity, longer hospital stay, and higher healthcare costs. The depth of anaesthesia has been theorized to influence the incidence of these complications, with less anaesthesia thought to reduce risk. This meta-analysis evaluated the impact of light versus deep anaesthesia on the incidence of POD and POCD, time to PACU discharge, and hospital stay among older surgical patients.

Although our pooled results demonstrate a consistent association between deeper anaesthesia and neurocognitive complications, these findings should not be interpreted to provide proof of a direct causal relationship since neurocognitive outcomes are associated with multiple perioperative factors including drug exposure, surgical stress, comorbidities, and intraoperative physiology.

Seven RCTs involving 1,897 elderly patients demonstrated that light anaesthesia reduced the incidence of POD by approximately half compared with deep anaesthesia (OR = 0.52; 95% CI: 0.41-0.66; P < 0.00001), with negligible heterogeneity across studies (I² = 0%), indicating consistent results.

These results concur with those of a multicentre RCT by Wildes et al. [23], who determined a lower incidence of POD in patients undergoing anaesthesia with lighter levels of anaesthesia guided by EEG monitoring. The study emphasized the prevention of deep planes of anaesthesia to prevent POD in older patients undergoing major surgery.

In three RCTs including 1,308 elderly patients [13,14,19], light anaesthesia was also associated with a significantly lower incidence of POCD compared to deep anaesthesia (OR = 0.55; 95% CI: 0.39-0.77; P = 0.0005). The low heterogeneity (I² = 15%) suggests homogeneous effects between studies.

This finding agrees with the meta-analysis of Wang et al. [23], whose result shows that light anaesthesia is associated with a lower risk of POCD among elderly patients. The research proved the influence of anaesthetic depth on postoperative cognitive function and recommended lighter anaesthesia to avoid the risk of POCD.

Our findings align well with recent expert guidance from the ASA. The 2025 ASA Practice Advisory on the perioperative care of older adults recommends expanded preoperative evaluation, including screening for frailty and cognitive impairment, as part of a broad strategy to reduce the risk of POD and POCD [24]. The advisory also suggests that dexmedetomidine may be considered to lower delirium risk while judiciously weighing its hemodynamic effects and emphasizes a need to evaluate and minimize the use of medications with central nervous system activity in this population. These recommendations reinforce the clinical relevance of titrating anaesthetic depth according to brain health strategies, particularly in elderly surgical patients [24].

Three trials [13,15,16] involving 1,476 patients showed that light anaesthesia significantly decreased time to discharge from PACU (MD = -9.43 minutes; 95% CI: -12.87 to -5.99; P < 0.00001). However, there was moderate heterogeneity (I² = 66%), mainly due to the small number of patients and wide confidence intervals in the study by Wong et al. [19].

These findings are supported by the research conducted by Chan et al. [13], who also concluded that patients under lighter anaesthesia had faster emergence and readiness for discharge from PACU. The research pointed out the benefits of lighter anaesthesia in terms of making postoperative recovery time shorter.

Three studies [13,15,16] including 1,109 patients examined the effect of anaesthetic depth on hospital stay. Overall, light anaesthesia showed a non-significant trend toward shorter stays (MD = -0.81 days; 95% CI: -1.85 to 0.24; P = 0.13), with high heterogeneity (I² = 89%). The study by Kunst et al. [16] favoured deep anaesthesia, which contributed to the observed heterogeneity.

The heterogeneity of this relationship emphasizes the effect of other variables, such as surgical technique and patient comorbidities, on the hospital stay. Further studies are required to determine the relationship between anaesthetic depth and hospital stay.

Another relevant aspect is that intraoperative immobility is largely achieved through neuromuscular blockade rather than hypnotic depth, and hence, differences in BIS targets do not necessarily imply differences in motor suppression [25].

Limitations

Despite the overall strength of evidence, several limitations should be noted. First, the included studies were limited to English-language publications. Second, while most studies were judged to have low risk of bias, two studies [13,14] had a high risk of performance bias due to lack of blinding, potentially influencing subjective outcomes like POD. One study [18] lacked clarity in allocation concealment, and others had small sample sizes or single-centre designs, which may limit generalizability. Additionally, in view of the variability in the depth targets and anaesthetic techniques across the included studies, our results are to be interpreted within the context of a general 'lighter versus deeper' anaesthetic strategy rather than strict BIS cutoff values. Moreover, moderate-to-high heterogeneity was observed in some outcomes, notably time to PACU discharge and length of hospital stay, although sensitivity analyses helped identify and resolve contributing sources of variability. Finally, some trials excluded patients post-randomization (for instance, protocol violations or missing data), which, although transparently reported, may introduce bias related to attrition. An important limitation is the inability to establish causality from the observed associations. The included studies, while randomized for BIS target, did not control several key confounding variables. The types and total doses of anaesthetic agents differed between groups, as maintaining a lighter plane of anaesthesia inherently requires lower drug administration. Similarly, the surgical insult, including the type, duration, and invasiveness of the procedure, was not standardized. It is possible that patients undergoing more complex operations were more likely to receive deeper anaesthesia and, concurrently, higher doses of postoperative opioids, a well-established risk factor for delirium. Consequently, the observed benefits of light anaesthesia may be partly mediated or confounded by these interrelated factors, reduced anaesthetic exposure, differential surgical stress, and lower postoperative opioid requirements. Our findings should be viewed as the effect of a bundled intervention, namely, BIS-guided lighter anaesthesia, rather than the isolated effect of the BIS number itself.

Although concerns regarding potential hemodynamic compromise with deeper anaesthesia or a possible increased risk of intraoperative awareness with lighter anaesthesia are frequently discussed in clinical practice, these outcomes were not consistently or systematically reported across the included RCTs. As a result, they could not be examined within the scope of this meta-analysis. These aspects represent important domains for future research, particularly in relation to balancing depth-guided titration with haemodynamic stability and awareness prevention strategies.

## Conclusions

This meta-analysis provides evidence that light anaesthesia produced specifically favourable outcomes across several domains, including a significantly lower incidence of POD (OR 0.52; P < 0.00001) and POCD (OR 0.55; P = 0.0005) and a significantly shorter PACU discharge time (MD -9.43 min; P < 0.00001), with a non-significant but favourable trend toward reduced hospital stay (MD -0.81 days; P = 0.13). In the context of the aging surgical population and the clinical burden of postoperative neurocognitive complications, these findings provide further support for considering BIS-guided lighter anesthetic techniques as a better-outcome strategy in elderly patients when clinically appropriate. Interpretation of these results should be placed within the context of identifying a strong association, but not a proven causative relationship, due to possible confounding from unmeasured variables such as total drug exposure and surgical stress. Further large-scale, multicentre RCTs are warranted to strengthen these findings and refine anaesthetic protocols for this vulnerable group.
